# Polar delivery in plants; commonalities and differences to animal epithelial cells

**DOI:** 10.1098/rsob.140017

**Published:** 2014-04-16

**Authors:** Urszula Kania, Matyáš Fendrych, Jiří Friml

**Affiliations:** 1 Institute of Science and Technology Austria (IST Austria), 3400 Klosterneuburg, Austria; 2 Department of Plant Systems Biology, VIB and Department of Plant Biotechnology and Bioinformatics, Ghent University, 9052 Gent, Belgium

**Keywords:** polarity, PIN proteins, epithelial cells, screen

## Abstract

Although plant and animal cells use a similar core mechanism to deliver proteins to the plasma membrane, their different lifestyle, body organization and specific cell structures resulted in the acquisition of regulatory mechanisms that vary in the two kingdoms. In particular, cell polarity regulators do not seem to be conserved, because genes encoding key components are absent in plant genomes. In plants, the broad knowledge on polarity derives from the study of auxin transporters, the PIN-FORMED proteins, in the model plant *Arabidopsis thaliana.* In animals, much information is provided from the study of polarity in epithelial cells that exhibit basolateral and luminal apical polarities, separated by tight junctions. In this review, we summarize the similarities and differences of the polarization mechanisms between plants and animals and survey the main genetic approaches that have been used to characterize new genes involved in polarity establishment in plants, including the frequently used forward and reverse genetics screens as well as a novel chemical genetics approach that is expected to overcome the limitation of classical genetics methods.

## 2. Introduction

Establishment and maintenance of cell polarity are one of the most fundamental topics in cell biology. Differences in cell polarization among different cell types enable them to form cell sheets that carry out the same function and result in the formation of various tissues and organs. At the cellular level, polarity can be described as an asymmetrical distribution of molecules, proteins, organelles or cytoskeletal strands along a particular axis [1]. Such organization of intracellular structures plays a crucial role during cell differentiation, proliferation, morphogenesis, intercellular communication and cell signalling. Cell polarity is of crucial importance in unicellular organisms that, thanks to asymmetrically distributed molecules inside the cells, are able not only to proliferate and move, but also to specify distinct cell sites to fulfil a different function. A canonical example of such an organism is the green alga *Acetabularia* that develops structures resembling organs of higher plants, such as rhizoids, stalks and cups [2]. In multicellular organisms, polarity plays an additional role in the communication between cells that is necessary for their cooperation and function as a whole organ. Although in both plants and animals cell polarity determines the integrity of the organism, in most animal cells polarity, once established, is retained throughout the lifespan, whereas in plants, owing to their sessile lifestyle, relocation of the plasma membrane (PM)-localized proteins between different polar domains plays an additional role in responding to the ever-changing environmental stimuli and in developmental plasticity. The mechanism that allows plants to align along the gravity vector involves the relocation of the PIN-FORMED3 (PIN3) auxin efflux carriers in columella root cells and endodermal hypocotyl cells to redirect the auxin flow [3,4]. Different life strategies between plants and animals are reflected in their distinctive development: although most animals shape their adult body plan already during embryogenesis, plants continue to develop their body architecture postembryonically and are able to rearrange it in response to environmental conditions.

In plants, virtually all developmental processes, such as embryogenesis, organogenesis, vascular tissue formation or regeneration, require the establishment or rearrangement of the polarity. Many aspects of this developmental flexibility are mediated by the plant hormone auxin that acts as a polarizing cue [5–7]. Through an asymmetric distribution between cells and the formation of local maxima and minima, auxin controls many developmental processes, such as embryogenesis [8,9], organogenesis [10–13], tropic growth [3,14–17], vascular tissue formation [18], root meristem maintenance [19–21] and apical hook formation [22]. An auxin concentration gradient in a tissue can be created by its localized synthesis or metabolism, but predominantly by polar auxin transport (PAT). PAT depends on polarly localized auxin influx and efflux carriers that guide the auxin flow direction [23]. Auxin efflux is carried out by a family of PIN proteins [24], most of which (PIN1, PIN2, PIN3, PIN4 and PIN7) are polarly localized on the PM, depending on PIN protein, cell type and developmental stage [25]. Already during embryogenesis, the localization of PIN1, PIN4 and PIN7 directs the auxin accumulation towards distinct parts of the developing embryo and results in the specification of the main apical–basal plant axis. After the first division of the zygote, auxin accumulates in the pro-embryo, which specifies the apical pole. At the globular stage, auxin starts to accumulate in the hypophysis where the future root pole will be established [8]. Besides PIN proteins, auxin transport is also facilitated by other components, such as AUXIN-RESISTANT1/LIKE AUX1 (AUX1/LAX) and MULTIDRUG RESISTANCE/PHOSPHOGLYCOPROTEIN/ATP-BINDING CASSETTE OF B-TYPE (MDR/PGP/ABCB), which are influx and efflux carriers, respectively [26]. The localization of these proteins depends on the cell type in which they are expressed; for example, in the protophloem, AUX1/LAX proteins are located on the apical part of the cells, whereas in the shoot apical meristem, they localize similarly to the PIN1 proteins on the basal part of the cells [27]. The ABCB auxin transporters, ABCB1/PGP1, ABCB4/PGP4 and ABCB19/PGP19, are mainly distributed equally at the PM; however, in root epidermal cells, ABCB4/PGP4 displays a more polarized basal or apical localization [28]. Unravelling the mechanisms of the polarization process at the cellular level is crucial for understanding how single cells are able to organize themselves in a polarized manner to form the tissues and organs of living organisms.

## 3. Comparison of vesicular trafficking and protein localization factors between polarized cells of plants and animals

**Figure 1.**
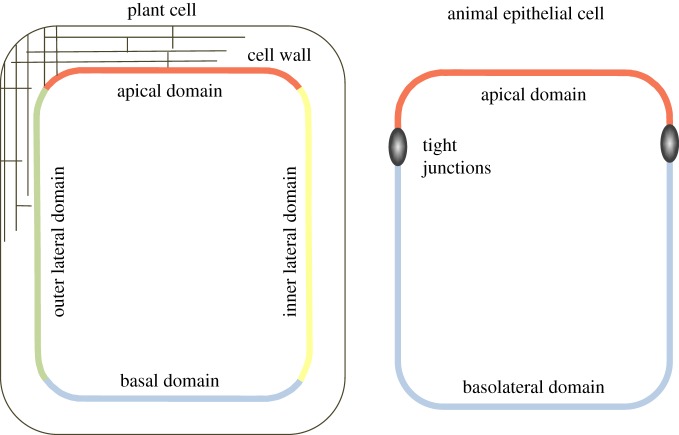
Schematic of polar domains in the plant epidermal and animal epithelial cells. Plant epidermal cells exhibit four polar domains, apical, basal, inner lateral and outer lateral, and are surrounded by cell walls. Animal epithelial cells exhibit apical and basolateral domains separated by tight junctions.

Eukaryotic cells share common cellular components that are involved in cell polarization, such as the endomembrane system, cytoskeleton, extracellular matrix/cell wall and molecular regulators of polarity (such as Rab GTPases). Nevertheless, the independent evolution of multicellularity in plants and animals resulted in the origin of specific executors and structures, such as cell walls in plants or tight junctions in animals, associated with the establishment and maintenance of polarity. In the animal system, the most remarkable polarity determinants (partitioning defective (PAR) and the Scribble and Crumbs complexes) serve as components to multiple effector pathways, including cytoskeleton formation, cell–cell junctions and cell membrane and cortex organization, ensuring formation and maintenance of polar domains as a consequence [29–33]. Plants have established their own polarization manner based on the activity of the Rho-like small G proteins, designated RAC/Rho of plants (ROP) GTPases [34], which are domain identity proteins. ROP GTPases are master molecular switches controlling cell polarization by regulating vesicle trafficking, interacting with cytoskeleton or working as domain identity proteins. Additionally, PIN proteins are important factors that induce their own polarity: they are auxin transporters, not regulatory proteins, and they need a polarized vesicular transport. Furthermore, the polar domains are differently organized in plants and animals (figure 1). In plant epidermal cells, four polar domains have been identified: the apical, basal, outer lateral and inner lateral, whereas in animal epithelial cells, only basolateral and apical domains can be distinguished separated by the so-called tight junctions [35].

### 3.1. Conserved trafficking cellular machinery and organelles

In both plants and animals, polarly localized proteins follow the secretory pathway from the endoplasmic reticulum (ER), through the *cis*- and *trans*-Golgi stacks, to the PM [36]. Proteins are transported between the intracellular compartments in vesicles that are formed by three classes of protein complexes: the coatomer protein complex II (COPII) guides the anterograde transport from the ER to the Golgi apparatus; the coatomer protein complex I (COPI) is crucial for retrograde transport from the Golgi apparatus to the ER and the intra-Golgi stacks [37,38]; and the adaptor protein (AP) clathrin complex encapsulates proteins during the transport between PM, Golgi apparatus and endosomes [39].

Sorting and polar targeting of newly synthetized proteins to the PM are better examined in animal systems. For a long time, only three main routes had been described for the secretion of polar proteins after exiting the *trans*-Golgi network (TGN). Proteins could be targeted directly to the apical polar and basolateral domains or, in some cases, apically localized proteins could follow an indirect route and go first to the basolateral domain from where they were transcytosed to the apical side [40,41]. The direct targeting to the polar domains was identified in the 1980s by biochemical and morphological studies that investigated the localization of different viral proteins in epithelial cells. After coexisting at the TGN, the influenza virus haemagglutinin and the vesicular stomatitis virus G protein were targeted directly to the apical or basolateral PM domains, respectively [42–44]. Recently, new experiments have demonstrated that the secretory pathway of some proteins can be more complex, encompassing the so-called recycling endosomes (REs) on the way from the Golgi apparatus to the PM. Although in animals the trafficking of newly synthetized, polarly localized proteins is well characterized, in plants this process is still unclear. In *Arabidopsis* cells, proteins are probably secreted in a polar manner [45]. The transcytosis of PIN proteins in plants has been shown [46,47] but it remains unclear whether this process assists the polar delivery in general or serves only for the repolarization after signals, such as gravity [48].

**Figure 2.**
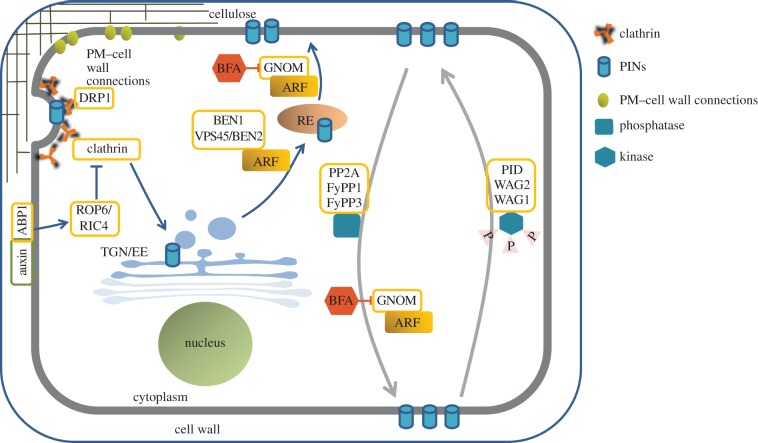
Intracellular trafficking and cellular requirements for polarization of PIN proteins. Auxin binding to its receptor ABP1 inhibits clathrin-mediated endocytosis (CME) through ROP6/RIC4 signalling. PIN proteins require the DRP1 function for CME. They are internalized to the TGN/EE and then follow the pathway to the RE that is regulated by BEN1 and VPS45/BEN2 ARF-GEFs. Recycling of PIN proteins from the RE to the PM is regulated by a GNOM-dependent mechanism. Control of apical and basal PIN targeting depends on the phosphorylation status of PIN proteins. PIN proteins are directed to the apical domain through phosphorylation by PID/WAG1/WAG2 kinases, whereas they are guided to the basal domain by dephosphorylation by means of PP2A/FyPP1/FyPP3 phosphatases. Basal targeting of PIN cargoes is controlled by GNOM. BFA, brefeldin A.

Besides the involvement of polar secretion in the cellular polarization, establishment and maintenance of the distinct polar domain is also regulated, in both plants and animals, by the constant polar recycling of the PM proteins. In epithelial cells, internalized proteins from the apical and basolateral domains localize to the apical and basolateral early endosomes (EEs), respectively, from where they can be recycled back to the PM, or targeted to the common recycling endosome that plays multiple roles in the protein-sorting pathway where common trafficking pathways intersect, such as recycling, secretion and transcytosis. Additionally, an apical recycling route encompasses the apical recycling endosome that is involved in basal-to-apical transcytosis and transport of newly synthesized proteins [49]. In plants, PIN proteins are internalized from the PM to the TGN/EE compartments and can further follow either the recycling route to the PM via hypothetical compartments, the REs (figure 2), or the degradation route to the vacuole via prevacuolar compartments that correspond to late endosomes in plants [23,50].

Polar cargoes derived both from secretory and endocytic pathways have to be sorted to the destination site. In epithelial cells, sorting of secreted proteins occurs mainly at the TGN, whereas REs sort mainly recycling proteins [51]. By contrast, in plants, the TGN/EE is the compartment in which secretory and endocytic routes merge [52,53]. Exocytosis is mediated by an evolutionarily conserved complex, the exocyst, which consists of eight subunits (Sec3, Sec5, Sec6, Sec8, Sec10, Sec15, Exo70 and Exo84). In plants and animals, the exocyst is responsible for vesicle tethering to the PM [54–57]. Constant trafficking of PM proteins is required for their proper polar localization. Although the trafficking mechanisms between plants and animals are similar, there are some main differences at the molecular level. The cellular trafficking machinery is better described in the epithelial system. Different types of sorting endosomes are distinguished in epithelial cells, whereas in plants the main sorting station is EE/TGN. In addition, more polarly localized proteins were identified in epithelial cells, allowing a better insight into dissecting the trafficking routes. However, in plant cells a lot of important information is still missing, such as the sorting mechanism of de novo synthetized PIN proteins.

### 3.2. Clathrin adaptor complexes

Endocytosis and exocytosis are highly dynamic processes [58] that are key determinants of the PM integrity and that regulate transport and signalling at the cell surface. Clathrin-mediated endocytosis (CME) has been shown to be involved in the recycling of polarly localized proteins in plants and animals [59,60]. Protein endocytosis from the PM to the endosomal compartment is initiated by recognition of the cargo-sorting signals by the adaptor protein-2 (AP-2) complex that recruits clathrin to form clathrin-coated vesicles. The AP-2 complex can recognize two specific peptide motifs in the cytoplasmic domain of transmembrane proteins, the tyrosine-based and the dileucine-based motifs, as well as post-translational modifications such as phosphorylation and ubiquitination [61]. Recently, the role of AP-2 in endocytosis has also been shown in plants [62,63].

From the five existing adaptor protein complexes, the AP-2 complex is implicated in CME [60,62,63]. As CME is the main endocytic route involved in the transport of PM components known in plants, it has a great influence on the polarization of PM proteins. However, in mammalian cells, also clathrin-independent mechanisms of endocytosis are known that regulate the PM composition. By contrast, other clathrin-dependent trafficking pathways have a tremendous impact on the polarization events. Analysis of clathrin knockdown mutants in Madin–Darby canine kidney cells has revealed that clathrin-mediated vesicle transport plays an essential role for the basolateral polarity without effect on the apical polarity [64]. Three of the adaptor protein complexes (AP-1, AP-2 and AP-3) have binding sites for clathrin [65], but the AP-3 function in polarized cell sorting has not been studied yet. The AP-1A and AP-1B complexes sort the proteins to the basolateral domain of epithelial cells, by recognizing the sorting signals and coating the proteins into clathrin vesicles [66]. The AP-1B complex occurs specifically in epithelial cells and differs from the ubiquitously expressed AP-1A complex in the µ1B subunit that is closely related to the µ1A [67]. The sorting signals for basolateral PM proteins are tyrosine and dileucine motifs that are similar to those recognized in CME [68,69]. Another sorting signal, a single leucine motif, has been characterized in the stem cell factor transmembrane growth factor, which is important specifically for basolateral sorting, but not for endocytosis [70]. Additionally, the AP-4 complex that does not interact with clathrin can recognize different basolateral sorting signals to mediate transport in epithelial cells, as confirmed by depletion of the µ4 adaptin that results in missorting of some basolateral proteins to the apical domain [71].

Although CME was well characterized in mammals and yeast, the genetic characterization of the clathrin involvement in this process has been identified only recently in plants. A first insight into the role of clathrin in CME was gained by immunolocalization of clathrin at different stages of vesicle formation [59]. CME was best described for PIN proteins as an important factor for their polar localization. When endocytosis is blocked by chemical inhibitors, PIN proteins at the PM spread laterally. Live-cell imaging and computational approaches revealed that laterally diffused PIN proteins that escaped from polar domains are internalized by clathrin-dependent endocytosis and via exocytosis are delivered back to the polar domain centre by superpolar recycling [72]. Characterization of the clathrin heavy chain 2 (*chc2*) mutants and dominant-negative clathrin heavy chain (CHC1) (*HUB*) showed defects in the bulk endocytosis and the recycling machinery of PIN proteins, with a defective polar targeting as a consequence [73]. Furthermore, mutations in different subunits of the AP-2 complex, such as the σ adaptin (*ap2σ*) or the µ adaptin (*ap2m*), result in impaired endocytosis and disruption of the polar PIN1-GFP localization during embryogenesis or of the PIN2-GFP localization in the male reproductive organ development [74,75]. Deficient PIN localization together with other developmental defects in clathrin and AP-2 mutants, such as reduced vegetative growth or impaired vascular patterns, which are reminiscent of defects in auxin signalling and transport, hint at an important role of CME in the polarization process. Another group of proteins involved in the CME process required for fission of clathrin-coated vesicles in mammals are dynamin-related proteins (DRPs). Although their function is not very well characterized in plants, DRP1A has been shown to interact with PIN proteins during CME at the cell plate. Examination of the *drp1* mutant phenotype confirmed the importance of these proteins for proper PIN1 distribution in dividing cells and of their role in auxin-mediated development [76]. Additionally, AP-1 is involved in intracellular protein sorting at the TGN/EE in the interphase and in protein delivery at the cell plate during cytokinesis [77–79]. AP-3 has been suggested to act in the transport from the Golgi apparatus to the plant vacuoles, but its function is still poorly defined [80,81] and its role in polarity has not been shown yet. Clathrin, together with the AP-2 complex, plays an important role in polarity maintenance, both in plants and animals. However, in plants CME serves as a main pathway for internalization and recycling of PM proteins, whereas in animals it is involved mostly in the regulation of basolateral trafficking. An additional important role in basolateral cargo delivery in epithelial cells is played by the AP-1 complex. In plants, other AP complexes have to be examined further to evaluate their role in polarization events.

### 3.3. Small GTPases

Small GTPases are a group of hydrolase enzymes implicated in a broad range of cellular signalling events. Of the many genes that code for GTPases and their regulators in plants and animals, some subfamilies are involved in polarization events, such as the Rho, Rab and ADP-ribosylation factor (ARF) GTPases. They regulate the vesicular trafficking between intracellular compartments by recruiting coat protein complexes to the vesicle formation sites, organizing the cytoskeleton and docking vesicles to the destination membranes. GTPase proteins constitutively cycle between their active GTP-bound and inactive GDP-bound conformations. Their activation is mediated by the guanine exchange factor (GEF) that stimulates the GDP-to-GTP substitution and the deactivation process by GTPase-activating proteins (GAPs) that promote the GTP hydrolysis and the return of Rho, ARF or Rab proteins to the GDP-bound form [82]. Additionally, the Rho protein has another regulator, the Rho GDP dissociation inhibitor (RhoGDI). During evolution, the Rho superfamily diverged into subgroups: characteristic for mammals and filamentous fungi, Rho, Rac and the cell division control protein CDC42; for yeast, CDC42 and Rho; and the plant-specific ROPs [83]. In metazoans and fungi, Rho and CDC42 are considered the major polarity organizers. In budding yeasts, a pre-existing budding scar provides a landmark for the formation of the next daughter cell, but CDC42 can polarize cells even in the absence of polarizing cues. CDC42 activated by its exchange factor polarizes actin filaments towards itself to the new bud formation site, enhancing the activated CDC42 accumulation to the same site and depletion from other cell sites [84]. In animal cells, CDC42 is necessary to polarize PAR proteins; it interacts with the PAR3/PAR6/atypical protein kinase C (aPKC) polarity complex and maintains tight junctions [85,86]. In general, in animals and yeasts, Rho GTPases influence the actin filament formation and regulate vesicle transport by actin polymerization targeting to the PM domains, where they deliver the proteins [87]. In the mammalian system, the secretory and endocytic pathways are regulated by the Rab family of small GTPases that play a role in the different steps of membrane trafficking, i.e. budding, delivery, tethering and fusion [88], but only a few of them might have a specific function in the basolateral and apical trafficking. The small GTPase Rab8 regulates the basolateral cargo delivery by interacting with the AP-1B complex and the exocyst-tethering complex, which is implicated in basolateral cargo delivery [89]. Besides basolateral polarization, Rab8 is also involved in apical protein localization in intestinal cells [90] and in de novo generation of the apical domain and lumen [91]. Another Rab GTPase, Rab10, together with Rab8, mediates cargo trafficking from the TGN to the basolateral surface of newly synthetized proteins [92], whereas Rab25 and Rab11 control the apical recycling in epithelial cells [93]. Different Rho and Rab proteins mark polar PM domains and regulate polar exocytosis by interaction with the exocyst complex. The first small GTPase found to interact with the exocyst was Sec4 in yeast [94]. In epithelial cells, basolateral exocytosis is controlled by Rab8, Rab10, CDC42 and RalA, whereas Rab8, Rab11 and Rabin8 (Rab8GEF) drive the transport to the cilium in the apical domain [95]. Another small GTPase, ARF6, regulates the CME in the apical and basolateral domains [96]; besides its function in endocytosis, it plays also an important role in actin cytoskeleton rearrangements.

Recent experiments have improved the knowledge on the involvement of ROP GTPases and their interactors in polarity establishment in plants. In some cell types, such as trichoblasts, the localization of ROP GTPases to specific membrane domains is determined by auxin. A local auxin gradient induces the ROP accumulation on the rootward end of trichoblasts, marking the future root hair growth position [97]. In pollen tubes, highly polarized growth that occurs at the very tube tip is governed by ROP1, which oscillates between an active and inactive form to maintain the optimal level for efficient tube elongation. Globally, RhoGDI and RhoGAP inhibit ROP1 to prevent lateral propagation from the apical cap. Furthermore, ROP1 influences the apical actin microfilament formation that drives the polar exocytosis of ROP activators and inhibitors, generating positive and negative feedback-regulatory mechanisms, respectively [34]. Furthermore, ROP6 and its downstream ROP-INTERACTIVE CRIB MOTIF-CONTAINING PROTEIN 1 (RIC1) effector are involved in CME of PIN proteins in roots, where they recruit clathrin to the PM. This process is regulated by auxin through the auxin-binding protein 1 (ABP1) that acts upstream of ROP6/RIC1 [98]. Recently, the PM-localized transmembrane kinase receptor-like kinases have been demonstrated to interact with the ABP1 protein at the cell surface and to activate ROP GTPases [99], which have been shown to be master regulators of the formation of interdigitated pavement cells where the locally activated ROP4 and ROP6 are responsible for lobe and indentation formation [100]. The interactor of the activated *ROP* gene, ICR1, mediates the interaction of ROP-Sec3 at the PM and is necessary to recruit PIN proteins to the polar domains [101].

Endocytosis of PIN proteins is not only mediated by clathrin [59,102] but is also dependent on the GNOM (GN or also known as EMBRYO DEFECTIVE30 (EMB30) or VASCULAR NETWORK7 (VAN7)) and GNOM-like1 (GNL1) ARF-GEFs [103,104], together with the Rab GTPase ARA7 [105]. The PIN1 proteins that are directed to the recycling route are controlled by the GNOM-regulated ARF GTPase [106]. GNOM consists of a Sec7 domain recognized by the fungal toxin brefeldin A (BFA) that inhibits GNOM-dependent exocytosis, resulting in the accumulation of internalized proteins in so-called BFA compartments, together with the TGN, and in the depletion of PIN proteins from the PM [106]. After a prolonged incubation with BFA or a genetic interference with GNOM, PIN1 proteins from the basal domain are gradually transported to the apical cell side, whereas apically localized PIN2 proteins in the epidermis are BFA insensitive, indicating the importance of GNOM in the basal PIN localization [46]. In addition to its role in the intracellular trafficking, GNOM is involved in the endocytosis process, together with another ARF-GEF, namely GNL1, and ARF-GAP, namely vascular network defective 3 (VAN3). Mutant analysis and localization of these factors at the PM confirmed the significant role of the ARF GTPase machinery in the endocytic process [103,104]. Besides the GBF class of ARF-GEFs that includes GNOM and GNL1, another class of BFA-inhibited guanine (BIG) nucleotide exchange proteins is also involved in intracellular trafficking, although it is still not well characterized. One member of this class, i.e. BFA-visualized endocytic trafficking defective 1 (BEN1)/BIG5/MIN7, has been found to be involved in BFA-induced internalization of basally localized PIN proteins: PIN1-GFP in the stele and PIN2 in cortex cells [107]. BEN1, together with BEN2/VPS45, functions in early endosomal trafficking, which is required for polar PIN localization [108]. Besides ARF and ROP GTPases, also other small GTPases, such as Rab GTPases, play a role in the regulation of vesicle trafficking and polar PIN localization. BFA-visualized exocytic trafficking defective5 (BEX5)/RabA1b is associated with trafficking and proper PIN polarization. BEX5 is localized to the TGN/EE compartment and is implicated in exocytosis and transcytosis processes of PIN proteins [109]. Small GTPases are crucial for many steps in endomembrane vesicle trafficking, such as vesicle formation, movement, tethering and fusion. However, owing to the divergence of the GTPases in evolution, they regulate distinct stages in vesicle trafficking and have a different impact on cell polarization processes in plants and animals.

### 3.4. Phosphorylation

Protein phosphorylation is a post-translational modification that occurs on serine, threonine or tyrosine residues and that is catalysed by kinase enzymes. The reverse process of phosphate groups removal is mediated by phosphatases. Besides other roles, the phosphorylation status of proteins in plants and animals serves as an intrinsic cue for polar cargo delivery.

In mammalian cells, phosphorylation plays an important role in polar cargo delivery to the PM. Two main kinases are involved in this process: the serine/threonine kinase LKB1/PAR4 that is activated by the bile acid taurocholate and, in turn, triggers the second AMP-activated protein kinase (AMPK). LKB1 additionally activates 11 AMP-related kinases, including the four mammalian PAR1 paralogues [110]. LKB1 has been described first as a polarity determinant in a genetic screen for mutants defective in cell divisions of early *Caenorhabditis elegans* embryos that were designated *partitioning defective* (*par*) [111]. After fertilization, the first asymmetrical cell division of the zygote is crucial for proper establishment of the polarity axis in the future embryo. In most of the *par* mutants, the first cell division is symmetrical, leading to the synchronous division of the daughter cells, with severe defects in cell specification as a consequence. Each of the six PAR proteins identified so far is distributed in a characteristic manner after the first asymmetric cell division, indicating that their role is crucial for the formation of anterior–posterior cell polarity [112]. That PIN protein sorting to the apical or basal domains relies on its phosphorylation status [113–117] could be demonstrated after study of the localization of distinct PIN proteins in the same cell type. In root epidermal cells, the ectopically expressed PIN1 was located on the basal cell side, in contrast to the apically localized PIN2, hinting at sequence-based determinants for polar PIN localization [118]. Sequence analysis and *in vitro* phosphorylation assays revealed that phosphorylation of PIN1 by PINOID (PID) kinase occurs in the central hydrophilic loop [119] on several serine residues [116,117]. An antagonistic function of the serine/threonine PID kinase and protein phosphatase 2A (PP2A) in the polar PIN trafficking was demonstrated by a genetic study of *pp2a* and *pid* mutants in embryo and root development. PID phosphorylates PIN proteins to direct them to the apical domain, whereas PP2A counteracts the PID activity and dephosphorylates PIN proteins, targeting them to the basal cell side [119]. Close analysis of the *pid* mutant phenotype with defective apical polarization revealed that the apical PIN2 localization was intact in root epidermal cells [120], implying that additional kinases are present that redundantly regulate the phosphorylation status of PIN proteins. WAVY ROOT GROWTH1 (WAG1) and WAG2 kinases, which belong to the AGC-3 kinases, phosphorylate PIN proteins, together with PID, predominantly at the PM, from where they are directed to the apical recycling pathway after endocytosis [115]. Mutations in *pid*, *wag1* or *wag2* lead to root meristem collapse and agravitropic growth [121]. Another kinase involved in the phosphorylation of PIN proteins is D6 protein kinase (D6PK). D6PK colocalizes with PIN proteins on the basal membrane of the stele, cortex and lateral root cap cells. The *d6pk* mutant was shown to be defective in auxin transport, but its exact role remains unclear [122].

PP2A phosphatase with its multiple regulatory (A and B) and catalytic (C) subunits produces various holoenzymes with distinct functions and properties. Analysis of the loss-of-function mutants of three PP2AA isoforms revealed abnormal cotyledon phenotypes and aberrations in early embryo developmental stages [119,123] that resembled embryos with defects in auxin transport [8] and the *pin1* and *pid* mutant phenotypes, implying a role for the regulatory A subunit in basal PIN localization. Three isoforms of the regulatory A subunit gene family together with the catalytic subunits phytochrome-associated serine/threonine protein phosphatase1 (FyPP1), its homologue FyPP2 and SAPS DOMAIN-LIKE proteins physically interact to form the PP6 heterotrimeric holoenzyme complex [121]. Genetic interference in *FyPP* genes by mutations or their dominant-negative versions results in an altered PIN phosphorylation level that causes a basal-to-apical shift of PIN1 in stele cells and of PIN2 in cortex cells [121]. Recent data also indicated that the catalytic subunits of the PP2A subfamily II, PP2A-C3 and PP2A-C4, redundantly regulate embryo patterning and root development and affect the PIN1 protein polarity [124]. In plants and animals, the protein phosphorylation status plays a crucial role in their localization to the proper polar PM domains. However, different kinases and phosphatases are involved in the phosphorylation process: the LKB1/PAR4 and AMPK kinases in animal cells and the PID, WAG1, WAG2 and D6PK kinases, together with the PP2A phosphatase and PP6 complex, in plant cells.

### 3.5. Cytoskeleton involvement in cell polarity

Actin filaments and microtubules are polar polymers oriented along the polarity axis and consist of actin subunits and tubulin heterodimers, respectively. The polarity of cytoskeletal structures results from the unidirectional association of the subunits that can polymerize and depolymerize in a fast manner, depending on changing polarity signals [125]. Trafficking of vesicles and polar deposition to the PM takes place along the cytoskeleton. The cortical cytoskeleton serves also as a scaffold structure that determines the animal cell shape, whereas the plant cell shape relies on cell wall and turgor pressure. Treatment of actin and microtubules with depolymerizing chemicals revealed that the cytoskeleton targets polarly localized proteins, such as PIN proteins [47].

In epithelial cells, the actin cytoskeleton plays a role in the vesicle assembly at the Golgi and endosomes and in the vesicle transport across the cytoplasm. Actin, together with actin-associated proteins (such as spectrin, ankyrin and myosin) and the actin-regulatory protein CDC42, which is considered a main polarity regulator in most eukaryotes, regulate the vesicle exit from the TGN to the basolateral domain. CDC42 is necessary for the polymerization of actin cables in a polarized orientation and, subsequently, in directional transport. Interruption of the CDC42 function by knockout mutation leads to a reduced transport from the Golgi apparatus to the basolateral domain and an increased trafficking to the apical domain [126]. Additionally, actin depolymerization results in transcytosis of the cargo vesicles from the basolateral EEs directly to the apical surface, omitting the REs [127]. In many epithelial cells, the microtubular orientation designates the apical–basal cue of the cell: microtubule minus-ends face the apical, and plus-ends face the basal domain. Microtubules together with microtubule motors are also involved in the vesicular sorting/transport from the TGN and endosomes to the apical PM [128].

The impact of actin on differentially localized PIN proteins in distinct cell types was checked after actin interference with latrunculin B. The experiment revealed an essential role for the actin filaments in both apical and basal cargo deliveries, but the apical targeting seemed to be more sensitive to actin disintegration [47]. Microtubules are essential both in plants and animals during cell division as well as in the interphase to maintain the general cell polarity. Disruption of the microtubule organization interferes not only with the general vesicle trafficking with cellular shape loss as a consequence, but also specifically with the polar PIN trafficking. After microtubules had been depolymerized with oryzalin, basally localized PIN proteins were mislocalized and shifted preferentially to the apical domain, whereas their apical localization was largely unaffected, indicating that, in contrast to actin filaments, an intact microtubule organization is needed for basal PIN trafficking [47]. Additionally, the same cargo can be transported by two different pathways, depending on the cell cycle phase. PIN1 trafficking in the interphase between PM and endosomes depends on actin filaments, whereas delivery to the cell plate during cytokinesis depends on microtubules [129]. In summary, both the actin and microtubule cytoskeleton are crucial for establishment and maintenance of cargoes at the polar domains, but further analysis is needed to dissect their role for specific cargos and their regulation.

### 3.6. Specific non-conserved polarity components: tight junction, Casparian strips and cell wall

In addition to similar components of the basic cellular machinery and the involvement of the cytoskeleton and clathrin adaptor complexes in the establishment and maintenance of polarity, there are also other structures that are specific either only for animals, such as tight junctions that serve as physical borders between apical and basolateral polar domains, or for plants, such as cell walls. A structure comparable to the tight junctions also exists in plants, but is present exclusively in the endodermis, namely the Casparian strips, which are belts made of specialized cell wall material that acts as an extracellular diffusion barrier [130].

In polarized epithelial cells, basolateral and apical domains are separated by tight junctions that form a mechanical barrier against diffusion events and regulate paracellular permeability, and as a consequence help maintain the unidirectional transport of macromolecules across the epithelial cells. Tight junctions consist of transmembrane and peripheral membrane proteins that interact with the cytoskeleton and form a protein complex involved in polarity and proliferation control through signalling transduction pathways. The tight junctions consist of a few main families of transmembrane proteins: occludin, claudins, E-cadherins and junctional adhesion molecules [131], among which E-cadherin is a crucial protein in cell–cell adhesion and cell polarization and is required for the tight junction orientation and lumen positioning. These proteins also promote basolateral cargo delivery to the cell–cell adhesion sites and lateral membrane domains [132]. Two evolutionarily conserved protein complexes, PAR and Crumbs, take part in the organization of the tight junctions and are involved in polarity establishment and maintenance in epithelial cells. The Crumbs/proteins associated with Lin Seven1 (PALS1)/PALS-associated tight junction protein module is linked via PALS to the PAR3/PAR6/aPKC module that helps in the establishment of tight junctions and contributes to the formation of the apical domain, whereas the DISCS LARGE (DLG)/Scribble/LETHAL GIANT LARVAE (LGL) module functions at the basolateral domain [133].

Until recently, no data had been provided for the connection between polarity maintenance of PIN proteins and the cell wall integrity. Such a link was suggested after the characterization of the *regulator of PIN polarity3* (*repp3*) mutant, which is affected in the ectopically expressed *PIN1* gene. The mutation responsible for the phenotype was localized in the gene coding for CELLULOSE SYNTHASE CATALYTIC SUBUNIT3/CONSTITUTIVE EXPRESSION OF VEGETATIVE STORAGE PROTEIN1 (VSP1)/ISOXABEN-RESISTANT1/ECTOPIC LIGNIN1 (CESA3/CEV1/IXR1/ELI1) [134]. CESA3 is part of the cellulose synthase complex that is localized at the PM and is responsible for the synthesis of the β-1,4 glucans, the building blocks for cellulose microfibrils [135]. Moreover, chemical disintegration of the cell wall and plasmolysis experiments revealed that proteins localized in the polar domains are attached to the extracellular matrix in a cellulose-dependent manner, preventing lateral protein diffusion [134]. The data obtained from the genetic analysis of the *repp3* mutant and a pharmacologic study also indicated that the cell wall is implicated in the process of PIN polarity maintenance [134]. 

## 4. Genetic approaches to dissect polarity in plants

To gain more insights into the process of polarity establishment and maintenance, it is important to characterize all proteins involved in these signalling cascades. Different methods are applied to find novel genes active in this process. One such method is the forward genetic screen, in which mutants with the desired phenotypes are mapped to find the causative mutation. In the reverse genetic approach, the gene function and its action in various processes are assigned by analysing the phenotypic changes after perturbation of the gene activity. Mutagenesis induction in the *Arabidopsis* genome can be achieved by using different biological and chemical agents, of which ethyl methanesulfonate (EMS) causes predominantly single base-pair substitutions.

### 4.1. Identification of polarity-defective mutants by morphological phenotypes

Several polarity-linked mutants were found in genetic screens based on morphological phenotypes, such as the *gnom* mutant with impaired basal PIN trafficking, the *pid* mutant with affected apical PIN distribution, and the *macchi-bou 4/enhancer of pinoid/naked pins in yuc mutants 1* (*mab4/enp/npy1*) with preserved PIN proteins at the PM in the polar domains. The *gnom* mutant was first discovered in a screen for mutants defective in pattern formation in *Arabidopsis* seedlings [136]. The loss-of-function *gnom* mutant displays severe phenotypes, including lack of roots, fused cotyledons, defects in vascular patterning and defective formation of the embryo axis [137]. Further analysis of GNOM function hinted at a role in embryo axis formation [138] and postembryonic development of *Arabidopsis* [139]. All the phenotypes of *gnom* mutants can be mimicked by application of a high dosage of auxin or PAT inhibitors, demonstrating the connection between GNOM and auxin transport. Further characterization of GNOM revealed that its action mechanism is the regulation of the basal PIN localization and that it is a crucial component in this process.

Another mutant with a key function in the polar PIN localization is *pid* [140], which has been isolated in a screen for mutants defective in inflorescence meristem formation. Besides the defects in floral organ development, the *pid* mutant is also impaired in cotyledon and leaf growth [141]. The defective bud formation in the *pid* mutant is similar to the *pin1* phenotype and the phenotype induced by the PAT inhibitors, indicating that both mutations play a role in the PAT [142]. Furthermore, characterization of loss-of-function and gain-of-function PID lines revealed that PID is implicated in the polar PIN localization [113].

Genetic analysis of the *laterne* mutant that displays a complete deletion of cotyledons pointed towards two phenotype-causing mutations: one in the *PID* gene and another one in the *MAB4/ENP* gene [143]. Through a detailed analysis of *MAB4/ENP* and other members of the *MAB4/ENP* subfamily, its polar localization at the PM and function in retaining of PIN proteins at the PM was validated [144,145].

### 4.2. Fluorescent marker-based forward genetic screen

An EMS-treated population of transgenic *Arabidopsis* plants with the PIN1-GFP marker was used in a forward genetic screen to identify new mutants defective in the accumulation and/or internalization of PIN1::PIN1-GFP into BFA compartments [107,108]. Mutants were screened using a fluorescence microscope to identify the desired subcellular phenotype. Three mutants were characterized and designated *ben1*, *ben2* and *ben3* (from BFA-visualized endocytic trafficking defective). They were defective in agglomeration of internalized PM proteins, but showed a different sensitivity to the aggregation of endosomes and the Golgi apparatus, hinting at their distinct role in intracellular trafficking. *ben1* has been identified as an ARF-GEF component from the BIG subfamily of early endosomal trafficking, AtMIN7/BIG5, with a defect in polar PIN1 localization, whereas *ben2* codes for the SEC1/Munc18 family protein BEN2/VPS45, a universal constituent of membrane fusion in eukaryotic cells [107,108]. The BEN2 localization in the early endosomal pathway differs from that of BEN1 and mutations in the *BEN2* gene modify the intracellular trafficking of PIN proteins.

### 4.3. Reverse genetic screen

Whereas the aim of forward genetics is to find the genetic basis of phenotypic features, reverse genetics looks for phenotypes that result from gene modifications. In reverse genetics, specific genes are disrupted to find their function by comparing the mutated gene phenotypes with the wild-type organisms. Different approaches are used in *A. thaliana* reverse genetics, such as gene silencing with RNAi or artificial microRNAs, which specifically target the gene of interest, or T-DNA and transposon insertional mutagenesis and targeting-induced local lesions in genomes, which randomly perturb the gene activity. Recently, new tools for targeted mutagenesis have been introduced in plants which use sequence-specific nucleases, such as zinc finger nucleases, meganucleases and transcription activator-like-effector nucleases [146]. In mammalian cells, the Rab5 protein plays a pivotal role in the internalization of PM-localized proteins. In plants, two genes homologous to Rab5, designated Ara7 and Rha1, are involved in endocytosis as well. Mutations in either gene do not display any phenotype, but the double mutant *ara7rha1* and the knockout mutant of its activator Rab5-GEF AtVPS9a, is embryo lethal. Characterization of the dominant-negative version of Ara7 (*DN-Ara7*), which is an inactive Ara7 form, specified its role in endocytosis and in PIN polarity establishment [105].

Genetic interference with the CHC by overexpression of its C-terminal part led to the dominant-negative effect notable by impaired PIN internalization and defective plant development and auxin distribution [59,73]. Further characterization of the loss-of-function *chc* mutant confirmed the previous observation of the involvement of clathrin in endocytosis.

### 4.4. Specific screens for PIN polarity components

**Figure 3.**
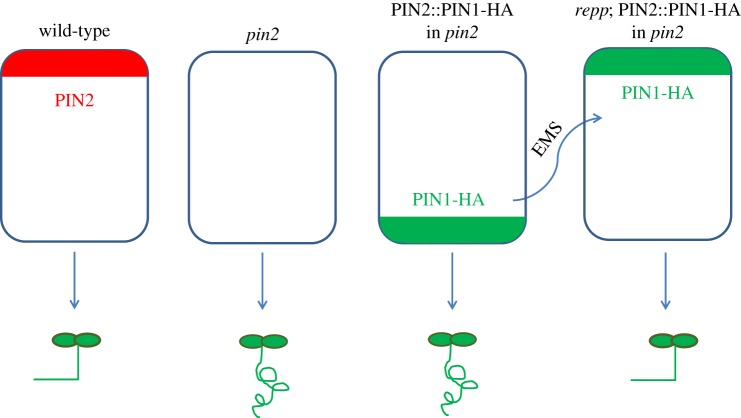
Design of a specific screen for PIN polarity components. PIN2 proteins localize to the apical side of epidermal cells in the gravitropic wild-type line. In the *pin2* mutant, PIN2 proteins do not occur, provoking the agravitropic phenotype. PIN1-HA is mislocalized in the epidermis to the basal cell side in the PIN2::PIN1-HA;*pin2* line, resulting in an agravitropic phenotype. Mutations in the putative PIN polarity regulators (*repp*) are predicted to restore the apical localization of PIN1 and, hence, the gravitropic phenotype.

Screening of mutants using the microscope by direct observation of the cellular PIN localization is very laborious and time consuming. To overcome these difficulties, it was necessary to translate the problem of polarity at the cellular level to a macroscopically visible phenotype that would be fast and easy to screen. Examination of the gravitropic response of the mutagenized transgenic PIN2::PIN1-HA line in the *pin2* mutant background provided the solution (figure 3). In wild-type plants, the apical localization of PIN2 in the root epidermis directs the auxin flow from the root tip to the top parts of the root, enabling root growth toward the gravity vector. In the PIN2::PIN1-HA line, the PIN1 proteins localize predominantly at the basal side of epidermal cells and, thus, do not rescue the agravitropic phenotype of the *pin2* mutant. Weak polarity mutants are mostly defective in PIN1 localization and exhibit a basal-to-apical polarity shift; hence, in polarity-defective mutants, the basal PIN1 proteins in the epidermis were hypothesized to be targeted to the apical domain, as macroscopically observed by the gravitropic growth restoration. Screening for mutants that respond to the gravity vector enabled the identification of the *repp3* mutant as a new candidate for the polar PIN localization [134].

### 4.5. Chemical genetic screen and chemical biology

Classical genetic approaches have greatly contributed to our understanding of polarity in plants. However, the plant genomes are genetically redundant, meaning that a cellular function can be encoded by more than one gene and, in the case of a mutation, the redundant gene can take over the function of the inactive one. As a result, some genes important for polarity might have been omitted in the classical genetic screens. Another limitation of classical screens is that mutations in genes that are crucial for polarity establishment and maintenance, which are the essential features of all living organisms, might be lethal. Biologically active small molecules can overcome these limitations, because they can be applied at different stages of plant development and at different concentrations, with a broad range of phenotypes as a result. Some small molecules can also target one specific protein, whereas others can affect entire protein families when a conserved region is targeted, thereby overcoming gene redundancy. Chemical genetic screens have already been used to dissect chemicals affecting cell wall synthesis [135,147], molecules inhibiting auxin signalling or transport [148–151], molecules inhibiting brassinosteroids synthesis [152] or those affecting the endomembrane system [153]. The endosidin1 (ES1) small molecule induced the selective accumulation of the auxin transporters PIN2 and AUX1 and the brassinosteroid receptor BRI1, but not other PM proteins, such as PIN1 and PIN7, providing a new tool to investigate recycling pathways [154]. Huge libraries of small molecules have been used in high-throughput screens as a novel tool to dissect the polarity process, although from the very beginning plant polarity research was conducted with small molecules, such as auxin analogues, antagonists and transport inhibitors. The synthetic auxin naphthalene-1-acetic acid was used in a forward genetic screen that helped characterize the *AUXIN-RESISTANT1* (*AXR1*) loci involved in the TIR1 and SKP1/CULLIN1/F-BOX PROTEIN (SCR^TIR1^)/AUXIN SIGNALLING F BOX (AFB)-based auxin signalling pathway. Other substances such as BFA or wortmannin are commonly used in plant research to interfere with specific trafficking routes. Wortmannin induces the accumulation of PIN2 on its way to the vacuole by affecting phosphatidylinositol 3-kinase [155]. Other chemicals, such as 1-*N*-naphthylphthalamic acid or 2,3,5-triiodobenzoic acid inhibit the PAT [156] and interfere with the auxin trafficking transporters, the PIN proteins and PGPs, possibly by targeting the actin cytoskeleton [107], but the exact mechanism has still to be determined.

To assess the mechanism of polar targeting in plants, libraries of small molecules can be screened for modifiers of the PIN polar localization by means of an innovative chemical genomics approach. In a first round, a high-throughput screen was carried out based on the ability of small molecules to inhibit pollen germination in tobacco (*Nicotiana* sp.) or interfere with polarized tube growth. Potential inhibitors of pollen germination were selected and tested on their effect on the polar PIN localization [157]. By this approach, a set of bioactive chemicals affecting the basal PIN localization or PIN trafficking is selected and characterized. To further identify targets and affected pathways of the small molecules, the genetic resistance or hypersensitivity to the chosen molecule in *Arabidopsis* has to be screened. Although a chemical genetic screen is more laborious than a classical one because even two screens have to be performed—one to find biologically active molecules affecting the desired pathways and another to find the target protein of the chemical—it is nevertheless expected that it will be an instrumentally new method to identify novel regulators of polarity in plants.

## 5. Summary

The generation of polarity involves a complex machinery of interacting factors, including ROP GTPases, the cytoskeleton, vesicular trafficking, mechanical tensions (not described here), the extracellular matrix and environmental signals. In animal epithelial cells, the main polarization factors are PAR proteins that are involved in the establishment of the anteroposterior axis of the developing embryo. They localize polarly in the cells and mutually regulate their polarity. Additionally, CDC42 plays a role in polarity establishment by interacting with the PAR6 protein and is implicated in the association of the PAR3/PAR6/aPKC complex with the cell cortex. In plants, ROP GTPases can be considered as main factors responsible for the polarization processes that influence the local polarization of actin and microtubules. ROP GTPases accumulate at the PM landmarks, such as the growing pollen tube tip. The active GTPase sites trigger the local polarization of actin and microtubules that serve as roads along which the vesicles can be transported to the place of destination.
